# Seasonal Activity Budget of Adult Baltic Ringed Seals

**DOI:** 10.1371/journal.pone.0002006

**Published:** 2008-04-16

**Authors:** Tero Harkonen, Mart Jüssi, Ivar Jüssi, Michail Verevkin, Lilia Dmitrieva, Eero Helle, Roustam Sagitov, Karin C. Harding

**Affiliations:** 1 Swedish Museum of Natural History, Stockholm, Sweden; 2 Estonian Fund for Nature, Tartu, Estonia; 3 Estonian Marine Institute, Tallinn, Estonia; 4 St. Petersburg State University, St. Petersburg, Russian Federation; 5 St. Petersburg Naturalist Society, St. Petersburg, Russian Federation; 6 Finnish Game and Fisheries Res. Institute, Helsinki, Finland; 7 Department of Marine Ecology, Göteborg University, Göteborg, Sweden; Stanford University, United States of America

## Abstract

Although ringed seals are important components in oceanic and fresh water ecosystems at high latitudes, little is known about how they exploit these harsh environments. Seasonal activity and diving behaviour of 19 adult Baltic ringed seals were studied by satellite telemetry. We elaborated an activity budget for ten months of the year, extending over the period from moult to the breeding season. Seals from three main regions showed explicit site fidelity and the distributions of animals tagged from different areas did not overlap, suggesting separate stocks. Both the mean duration and the mean depth of dives peaked in June and July. Seals spent 70% (females) to 85% (males) of their time diving in June and July which decreased to 50% in late autumn. Less than one percent of dives exceeded 10 min in females, while 10% of male dives lasted longer than 10 min in June to September. Less than one percent of dives lasted for more than 25 min. Both females and males were most active during day time and hauled out predominantly during the night. Activity patterns during the summer are suggested to be correlated to energy accumulation and prey availability. The information on seasonal activity budget is crucial for developing population energetic models where interactions between ringed seals and other trophic levels can be evaluated.

## Introduction

Ringed seals (*Phoca hispida*) are the most numerous of all seal species in high arctic areas [1], and have thereby an important role in the ecosystem [2]. They constitute the main prey for polar bears (*Ursus maritimus*) and they forage on a number of fish and squid species, but also crustaceans are important food items [2]. However, most information on the ecology of ringed seals have been based on data collected during the phase of their annual cycle when they are associated with the ice, i.e. the pupping and moulting seasons [1]. The knowledge about ringed seal movements and behaviour in the open water season was until recently limited to information collected on hunted animals [3], but new opportunities appeared with the development of advanced dive recorders, where data could be relayed via satellites [4]. This technology has made it possible to study the movements and diving behaviour of seals within and outside the breeding and moulting seasons as well as during the dark polar night [4]–[9]. However, previous investigations carried out in Arctic ringed seals have mostly been based on young animals, and therefore knowledge of how adult ringed seals exploit these extreme ecosystems is still fragmentary.

Baltic ringed seals (*Phoca hispida botnica*) are landlocked within the Baltic basin and form a discrete population [10]. Although having been an important resource for coastal communities for centuries, there is virtually no information on the ecology of this formerly abundant species [11]. We here quantify the activity budget for adult Baltic ringed seals, emphasizing seasonal changes in behaviour, and discuss the results in relation to prey availability and energy accumulation.

## Results

The main focus of this study was to construct an activity budget for adult Baltic ringed seals, which has three main components: proportion of time hauled out on land, time spent at surface, and time spent diving. Detailed information on the seasonal and diurnal patterns in these components is given under separate headings. Seals from the three main areas, the Bothnian Bay, the Gulf of Finland and the Estonian coastal waters, were tagged with satellite transmitters and data was collected on horizontal movements and diving behaviour.

### Distribution

**Figure 1:**
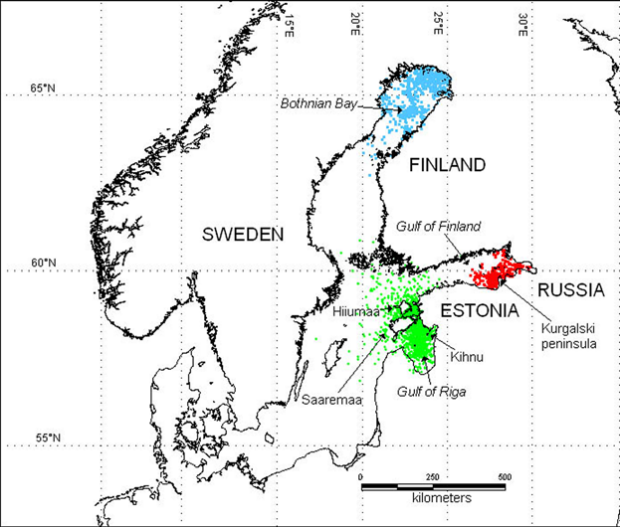
The Baltic Sea area with names mentioned in text. Locations of adult ringed seals tagged with satellite transmitters in the Bothnian Bay (blue, 345 locations), the Gulf of Finland (red, 178 locations), and Estonian coastal waters (green, 812 locations). There was no overlap in the distribution of seals tagged in the different areas.

Data on positions of tracked seals from the Bothnian Bay (345 locations of class 0 to 3) covered the period from November to March, and during this period the ringed seals were strictly found in the ice-covered areas in the north. None of five seals moved out of the Bothnian Bay (Fig. 1). The larger data set from Estonian coastal waters (10 seals, 812 locations) and the Gulf of Finland (4 seals, 178 locations) covering the period from mid May to mid March, indicate a similar behaviour, but also permits a more detailed analysis. In late May the seals left the shallow areas and most “at sea locations” during June and July were over deeper waters (>20 m) in the Gulf of Riga, and the Gulf of Finland. The positions from the deep off-shore waters were from the summer period, and in September and October all seals moved towards the main coast line. In December and January seals tagged in Estonia moved from the straits to the northern parts of the Gulf of Riga. This movement to the south coincided with the advancement of the fast ice edge in the straits. In years when the shallow Estonian coastal waters were covered by fast ice in February, none of the seals remained in the area. The males tagged in Russia showed a similar pattern and moved to the known breeding ice in the north-eastern part of the Gulf of Finland in November, and mainly stayed there through February, whereas both females stayed in the open waters of the Southern part of the Gulf. A more detailed analysis of seasonal movements and habitat selection will be given elsewhere.

### Diving behaviour

Data for all males will be analyzed in the same context since there were no significant differences in main patterns among individuals. However, the diving behaviour of females in the Bothnian Bay deviated substantially from females in the two southern areas, and are thus treated separately.

#### Depth of dives over the year in the two southern areas

**Figure 2:**
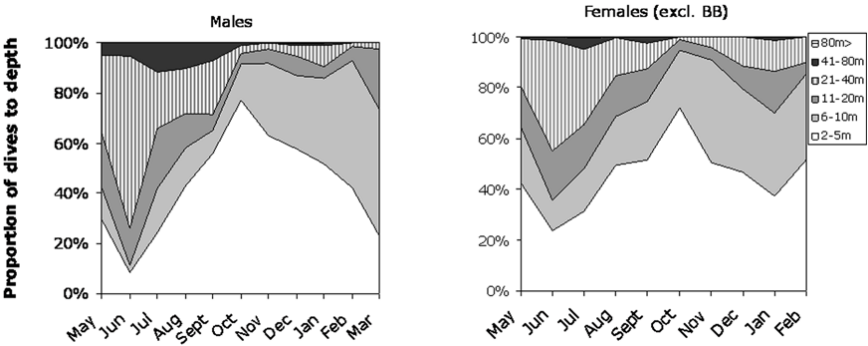
Proportions of dives to depth intervals from 2–5 m to more than 80 m of adult ringed seals. Females tagged in the Bothnian Bay (BB) not included. More than 90% of male dives were deeper than 5 m in June, which changed to 25% in October to again increase to about 80% in March. A similar flux was evident in females, but the dives were generally shallower than for males.

Most dives of females were shallow (<10 m) over the year, but numerous dives were also found in the >20 m–40 m bin (Fig. 2). In May 37% of female dives were deeper than 10 m, but the proportion increased to 64 and 57% in June and July, respectively (Fig. 2). Also the frequencies of the deepest dives (>80 m) peaked during this period of the year. In late summer and in the autumn deeper dives became scarce, and in October only 7% of dives were deeper than 10 m. This proportion increased gradually during the winter and in January 31% of the dives were deeper than 10 m. Males showed basically the same pattern as found for females. The major difference was that the frequencies of deeper dives in June and July were higher for males compared to females. During winter the dives of the males were even shallower than for the females.

#### Duration of dives

**Figure 3:**
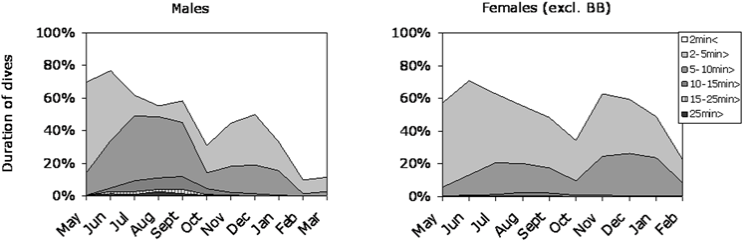
Duration (min) of dives of Baltic ringed seals (excluding females from the Bothnian Bay). About 70% of male dives lasted longer than 2 min in early summer, which decreased to 30% in October. An increase to about 50% was seen up to December, after which dive durations decreased markedly up to March. A similar pattern was seen in females, but dives lasted longer in December and January. About 10% of male dives lasted longer than 10 min in the period from June to September, compared with 0.8% for females.

For the two southern areas the duration of dives also showed strong seasonal variations for both sexes. In females, dives longer than 2 min decreased from 70% in June to less than 40% in October, after which the proportion of longer dives increased again between November and January (Fig. 3). However, in February about 80% of dives were shorter than 2 min. The pattern for males was basically similar, although male dives lasted longer in the summer and were shortest in February–March. Frequencies of the longest dives peaked in the summer (June to August) for both sexes, although only 1.7% of male dives and 0.15% of female dives lasted for more than 25 min.

#### Time at depth (TAD)

**Figure 4:**
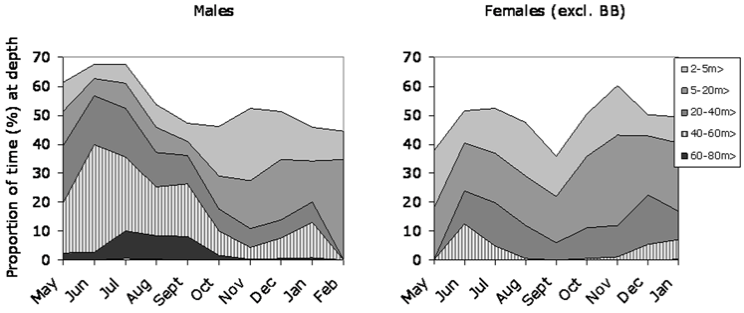
Proportion of time spent diving in different depth intervals over the year in the Gulf of Finland and Estonia. The total time diving deeper than 2 m is given by the border between the white and shaded areas.

In June and July the mean dive time exceeded 50% in females and was close to 70% in males. Slightly less time was spent diving in early autumn for both sexes, but it again approached or exceeded 50% in late autumn and winter (Fig. 4). Also here it is evident that males spent more time diving at greater depths, especially in the summer as compared with females. Males spent 7.5% to 9.5% of their time deeper than 60 m in July to September whereas females spent less than 0.01% exceeding that depth in the same period.

#### Maximum depth of dives

**Figure 5:**
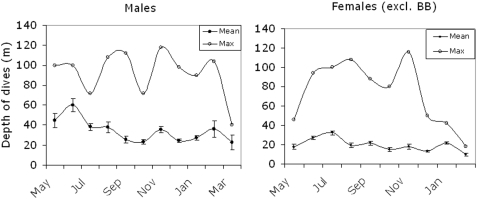
Maximum depths of dives from status messages given as maxima for each month and average monthly maximum values. Standard errors indicated by bars.

The reliability of the depth sensors was confirmed, since none of the recorded dives of ringed seals exceeded the maximum depth (120 m) of the sea bed in all three areas of the Baltic. The maximum depth of dives, over the previous 24 hour period, indicated that both male and female maximum dives were about 100 m in most months of the year. However, these deep dives were relatively rare, since the average daily maximum dive depths were about 40 m for males and 25 m for females. Monthly maximum depths were relatively shallow between December and February for females and in March for males (Fig. 5).

#### At surface time

**Figure 6:**
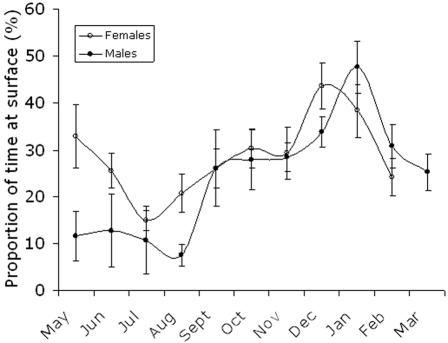
Seasonal changes in proportions of time spent at surface for adult ringed seals of both sexes in the Gulf of Finland and Estonia. N = 330 for males and 350 for females. Standard errors of estimates given.

The time spent at surface over the 6-hour period prior to transmission was given in the status messages (see Material and Methods). Males spent about 10% at surface from May to August, 25% in February–March and 49% in January, whereas females spent 16–35% at surface between May and August, 25–30% in the autumn and peaked in December (45%) (Fig. 6).

#### Time spent on land or ice

**Figure 7:**
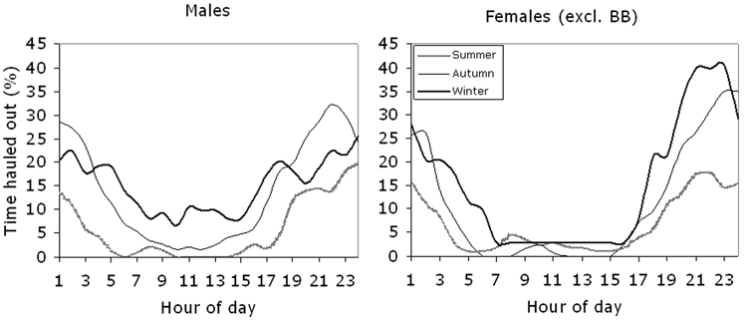
Diurnal haul-out behaviour of male and female ringed seals in the Gulf of Finland and Estonian coastal waters. Data from “Timelines” pooled for main seasons: Summer (May–Aug), Autumn (Sep–Nov) and Winter (Dec–March).

Estimates of time spent hauled out on land was provided by two partly independent alternatives; Timelines and the SLR (“dry & wet” transmissions). Timelines provide fine-scale data on the proportion of time spent in water over a 24 hour period, but only the last six transmitters deployed had this option. As shown by time-line data, seals of both sexes spent more time at sea during the light hours of day in all seasons, and they hauled out predominantly at night (Fig. 7). In winter (Dec–Feb) females hauled out more than 40% of their time during the night, compared with 25% of the time for males.

**Figure 8:**
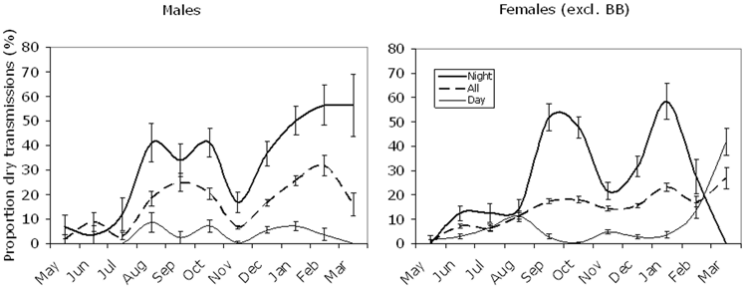
Seasonal changes in diurnal haul-out behaviour as given by the proportion of “dry” transmissions. Data given for night (2100-0300) day (0900-0300), and mean values for all hours of day (All). Standard errors of estimates given by error bars. N = 7066 for females and 3061 for males.

Complementary data on haul-out patterns was provided from the ratio of dry/wet transmissions (SLR), which was available from all transmitters. Also here it is evident that, over most of the year, both sexes spent nearly all their time in the water during the day (Fig. 8), a pattern which changed dramatically for females in the pupping season in February and March, when females seem to haul out 40% of their time during the day. Males spent up to 40%, and females up to 50%, of the night on the reefs in August and September. The nocturnal haul out time decreased substantially for both sexes in November, but increased again in January, when 60% of the time was spent out of water. In February and March night-time haul-out decreased substantially for females but not for males.

Comparing the data set from timelines (TL) and the SLR for animals that provided both data sets showed that the SLR data gave higher point estimates on the proportion of time hauled out. According to the SLR, mean percentage hauled out for males was 15.5% (SD 8.5%) in June–January compared with 10.8% (SD 7.5%) given by the TL. However, this difference was not statistically different (chi-square: p = 0.98). Females hauled out 10.7% (SD 6.4) according to the SLR and 9.7% (SD 6.2) according to the TL over the period May to November. Also in this case the difference was not statistically significant (chi-square: p = 0.99). We will use the SLR data to estimate the proportion of time hauled out since this data is available for all units.

#### Activity budget of adult ringed seals

**Figure 9:**
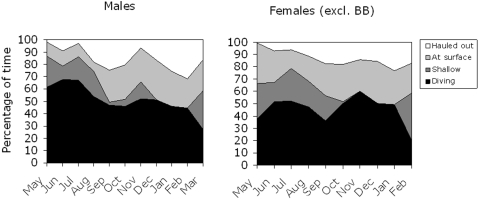
Activity budget for male and female ringed seals in the Gulf of Finland and Estonian coastal waters (pooled data). The proportion of time diving deeper than 2 m, time spent at surface, and time hauled out from figs 6, 8, and 11. “Shallow” denotes dives in the interval 1–2 m, and is given by 100% minus the sum of all other activities.

The sum of time spent diving, at surface and hauled out should add up to 100%, but since we defined the time spent diving as deeper than 2 m (Fig. 3), we lack information on the time spent in the depth interval from 1 m and up to 2 m. Assuming the difference between the sum of all other activities and the total amount of time was spent in this depth interval, the total activity patterns from moult to breeding can be estimated (Fig. 9). In males, the total time submerged was about 85% in May to July, which decreased to about 50% in September and October after which they spent 50 to 60% of their time under the surface up to March. Females spent about 70% of their time submerged from mid May to August after which a similar proportion was spent under water as in males. The main difference was seen in the summer when males spent about 10% of their time at the surface compared with 20 to 40% in females.

### Activity budget for females in the Bothnian Bay

**Figure 10:**
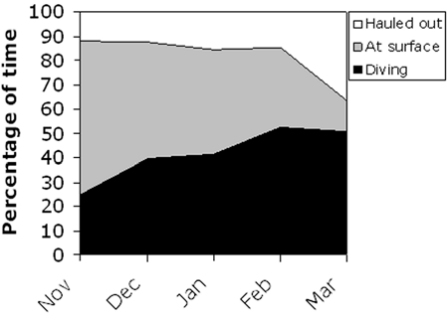
Activity budget for female ringed seals in the Bothnian Bay. Data pooled for four animals. Percentages of time diving (>2 m) extracted from time-at-depth data, and proportions of time hauled out from the “SLR”.

Data is only available for months November to March in this region, and we summarize the activities of females in the Bothnian Bay in Fig. 10. Females in this area dived deeper in late autumn and winter than both females and males in the two southern areas (chi-square: p<0.0001 in both cases). Here 13% of dives in January were deeper than 40 m, a pattern maintained over the winter since 17% and 23% of dives exceeded 40 m in February and March, respectively. A significantly different pattern (chi-square: p<0.0001 compared with both males and females in the southern areas) was also seen in the duration of dives where only 52% (Jan) to 64% (Feb) of dives in the winter were shorter than 2 min. Here 27% and 16% of dives lasted longer than 5 min in January and February, respectively.

The TAD data showed that the percentage of time spent diving deeper than 2 m increased from 25% in November to about 50% in February and March (Fig. 9). The proportion of time hauled out (SLR data) was 12% in November and increased to 15% in February, after which a substantial increase to 38% is suggested to March (Fig. 9). The time spent at surface seemed to decrease from 60% in November to 12% in March.

#### Weight gain during the open water season

**Figure 11:**
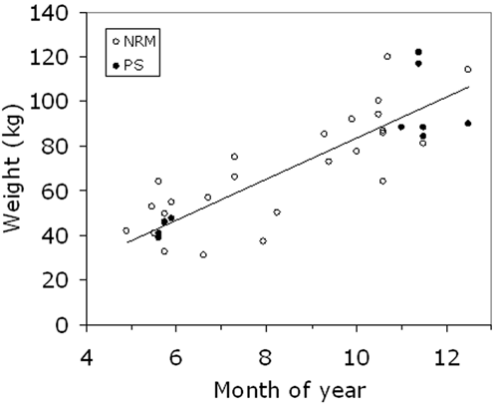
Weight of ringed seal females caught in the open water season. Filled circles refer to data from this study (PS), and open circles are data compiled from the seal data base at the Swedish Museum of Natural History (NRM). A linear regression of pooled data (y = 9.16x−7.97, R^2^ = 0.83) suggest that ringed seal females gain 9.2 kg per month from May up to December.

Pooling data from our study animals and data available from the Swedish Museum of Natural history shows that the mean weight of ringed seal females after the moult in May was 46.1 kg (SD = 8.3 kg) and a linear increase at 9.2 kg per month is suggested up to December when average females weighed 98.1 kg (SD = 16.6 kg) (Fig. 11.).

## Discussion

### Spatial distribution

Tracked animals showed explicit fidelity across the entire study period, and there was no overlap in the distributions of seals tagged from the three main areas. The suggested separation into three stocks has important implications for current and future conservation strategies, especially since only the Bothnian Bay component of the Baltic ringed seal population shows a weakly positive trend in abundance [11]. The two southern stocks are likely stabile or declining as a consequence of failed reproduction due to lack of suitable ice for breeding over the past three decades [11]. Strong spatial fidelity can be expected to result in very limited net migration of ringed seals to the southern areas, which should result in rapid declines in population numbers if the current high winter temperatures persist in the future.

### Ringed seal activity budget

Based on the large data sets from the 19 SDRs, and taking advantage of the variety of options of the devices, we constructed a detailed activity budget for adult Baltic ringed seals. There was an evident seasonal component, where diving activity peaked in June and July for both sexes, whereas a larger proportion of time was spent hauled out in the fall and in the winter. This pattern was seen in all types of data collected, which mainly coincides with results from earlier studies from other areas [6], [7], [9].

In the elaboration of the activity budget, we relied on the most extensive data sets for proportion of time hauled out, the SLR (Fig. 11). We compared estimates of haul out time based on SLR data and TL data for the six transmitters that provided both options, and found no significant differences, although point estimates suggested SLR data to give higher estimates compared with time lines. This is contrary to findings by Born *et al.* [7], where time-line data provided 1.4 times greater haul-out times as compared with SLR data. This difference might be linked to different programming of units.

### Diel activity and prey distribution

While we found substantial differences in diurnal haul-out and diving activities in all measured parameters, no such differences were revealed in NW Greenland [9]. However, in an earlier study from the same area it was shown that all seals were more active in the upper 50 m during the day in October–November, but no diurnal differences were detected earlier in the season [6]. One possibility that could explain this difference between Arctic and Baltic ringed seals could be linked to the midnight sun affecting prey behaviour and distribution, or general differences in prey behaviour.

Baltic herring (*Clupea harengus*) is one of the most important prey species of Baltic ringed seals [12], [13]. In the summer months, the Baltic herring undertakes diurnal vertical migrations, where they are close to surface at night, and close to the sea floor during the day [14]. Since ringed seals are suggested to forage mainly during the day (this study), Baltic ringed seals are expected to be more active in deeper waters during the day, as also indicated in our data. Such a pattern was also seen in Baikal seals, where diving activity was found to be significantly higher during daytime over the whole study period [15], and it was suggested that the diurnal differences were linked to vertical movements of prey [15].

### Differences between sexes

We found that adult males dived deeper and that dives lasted longer than in females especially in June and July in Estonian coastal waters and in the Gulf of Finland. The diving capacity of ringed seals has been shown to be positively correlated with body size [5], [16], [17]. Therefore, a part of the difference could be caused by size differences between the sexes, since the mean standard length of tagged females was 119.7±7.1 cm (SD) compared with 124.5±5.0 cm (SD) for males in the two southern areas combined. This could to some extent explain the deviating diving behaviours of females in the Bothnian Bay where the mean standard length of females was 128.8± 4.2 cm (SD), although differences in bathymetric conditions may play a major role. This highlights the problem of achieving samples that are representative of the population, and it is unlikely that low numbers of individuals will reflect the behaviour of a population.

### Vertical and horizontal patterns of movement

To be able to utilise resources in the environment, seals are forced to move between feeding areas and suitable shore or ice sites for hauling out and breeding [18]. For most Arctic and Antarctic seals, the ice serves as a platform for rest, reproduction or moult [1]. However, the most suitable haul-out sites for e.g. pupping, might not coincide with optimal foraging areas [19]. If haul out sites are surrounded by waters with low prey concentrations, seasonal migrations of seals [19], [20], [21] and diving patterns might be greatly influenced by the spatial distribution of foraging areas [22]. Seasonal horizontal movement patterns have been linked to changes in diving performance in a number of phocid seals such as grey seals [20], and harbour seals [18], [20], [21]. We envisage that the seasonal variation in diving behaviour of the Baltic ringed seals also is affected by spatial and temporal variation in prey distribution and access to haul out localities.

### Conclusions

The high degree of spatial fidelity can lead to severe consequences for ringed seals in the southern distribution areas (the Gulf of Finland and the Estonian coastal waters), where breeding has failed repeatedly over the last decades due to lack of breeding ice. Management actions in the Northern stock (the Bothnian Bay) can therefore affect the future of the entire Baltic ringed seal population.

All estimates of diving activity in adult Baltic ringed seals peak during the summer months, when seals are indicated to forage intensively. They double their body weight over the period from May to November, when many ringed seals exceed 100 kg. Diving patterns are suggested to be linked to the horizontal and vertical distribution of their main prey, the Baltic herring. Diving activity is substantially greater during the light hours of day, and seals haul out predominantly during the dark hours of the night, a pattern that changes in the breeding season for females. It is also suggested that the diving activity is affected by the size of the animals, since heavier animals dive deeper and longer compared with smaller individuals.

The information given here is essential for future elaborations of energy budgets of ringed seals, permitting analyses of the role of the species for the fishery and other components in the ecosystem. Future research should in that context focus on achieving data on main prey species, e.g. by fatty acid analyses, but also on more detailed studies on the diving energetics

## Materials and Methods

### Study area

The Baltic Sea (Fig. 1) is a brackish water system, where the salinity of the surface water shows a cline from 0.2% in the northern part of the Bothnian Bay to about 1.0% in the south, the Gulf of Finland approximately 0.4%, the Gulf of Riga about 0.5% (Fig. 1, [23]). The deepest areas (>200 m) are found in the Baltic proper (the area south of Åland, excluding the bays), whereas the mean depths in the Bothnian Bay and the Gulf of Finland range between 20 and 40 m. The maximum depths in those areas are 120 m. The Estonian coastal waters are dominated by large shallow areas, where depths seldom exceed 10–15 m. Deeper areas are found in the southern parts of the Gulf of Riga (50 m), and in the Baltic Proper next to the islands Hiiumaa and Saaremaa (120 m, Fig.1, [23]).

Ice formation normally starts in late November in the north and east, and the maximum ice coverage occurs in late February to early March. During average winters fast ice cover the Gulfs of Bothnia, Finland and Riga as well as the northern parts of the Baltic proper (Fig. [1], [24], [25]). In “mild” winters [24], [25], which dominated the study period, only the inner parts of the bays are covered by fast ice.

### Study population

The Baltic ringed seal population exceeded 180,000 animals in the beginning of the 20^th^ century, but decreased dramatically to 25,000 before 1940 as a consequence of an extermination campaign, predominantly administrated by Swedish and Finnish authorities [11]. A further decline to about 5000 animals in the mid 1980s has been attributed to environmental pollution [26], [11], which led to high levels of sterility in ringed seal females [26], [27].

The first winter surveys started in the early 1980s in the Bothnian Bay, when the hauled out population was estimated at about 3,000 seals [28]. Estimated numbers on ice dropped to 2200 in 1988, after which an increasing trend at 5% per year has been documented. [29]. The first systematic surveys in the Gulf of Finland and the Gulf of Riga, were conducted in 1996, and the hauled out populations were estimated to 250 and 1500 seals respectively [29]. Consequently, the total hauled out population in the entire Baltic Sea encompassed about 5,500 in 1996, suggesting a total “true” population size of about 10,000 animals since the hauled out fraction during moult is estimated at 57% [9], [29]. After 1996 the southern stocks have been stable or slowly declining [29].

The main pupping season for the ringed seal in the Baltic occurs between mid February and early March, followed by the annual moult, peaking between 20 April and 7 May [28], which is considerably earlier as compared with Arctic ringed seals [2].

### Tagging

Using commercial seal nets (Hvalpsund Nets A/S), ringed seals were caught in Estonian coastal waters (n = 10), the Bothnian Bay (n = 5) and the Gulf of Finland (n = 4) over the period 1994–1999 (Table 1). We caught eight seals in the autumn and 11 in the spring after moult (Table 1). We restrained them and a patch of fur between the shoulders of a seal was cleaned with alcohol, whereafter a satellite transmitter was attached with epoxy glue. Standard body length and weight were measured. A total of 13 females and six males from the three areas were tagged in this way (Table 1).

**Table 1:** Adult ringed seals at the Estonian west coast, the Bothnian Bay (BB) and the Gulf of Finland (GOF), which were equipped with Satellite linked Dive Recorders (SDRs).

Seal ID	Weight (kg)	Length (cm)	Area^2^	SDR No	Deployed	Last transm.	Transm. days
M1^1^	95.5	117	Estonia	20158	07-11-94	09-01-95	63
F2	88.5	128	Estonia	20165	07-11-94	25-02-95	110
M3	88.5	122	Estonia	20164	10-11-94	12-03-95	122
F4	90.0	126	BB	20163	17-12-94	24-03-95	96
F5	46.0	116	Estonia	3965	26-05-95	18-01-96	237
F6	41.0	116	Estonia	20170	19-05-96	23-01-97	250
F7	45.5	113	Estonia	20161	22-05-96	30-12-96	223
F8	46.0	113	Estonia	20159	22-05-96	03-02-97	258
F9	47.5	120	Estonia	20156	22-05-96	25-12-96	218
F10	117	127	BB	6338	05-11-96	02-02-97	89
F11	122	135	BB	6339	05-11-96	08-01-97	63
F12	84.0	127	BB	20695	13-11-96	24-03-97	131
M13	89.0	130	BB	20694	13-11-96	23-01-97	69
M14	51.0	123	Estonia	20156	18-05-97	14-02-98	272
F15	39.0	113	Estonia	29159	21-05-97	30-01-98	254
M16	75.0	130	GOF	20160	09-09-98	24-01-99	134
F17	74.5	128	GOF	20170	06-06-98	11-01-99	219
M19	75.0	125	GOF	6338	25-05-99	28-01-00	248
F20	80.0	130	GOF	20170	03-06-99	12-10-99	131

1 The sex of the animals is indicated by M or F in the first column.

2 Abreviations: the Bothnian Bay (BB) and the Gulf of Finland (GOF).

### Type of data collected

We used 0.5 watt Satellite Linked Time Depth Recorders (SDR) (Wildlife Computers, Seattle, USA) of Type 3. The first nine units (M1 to F9, Table 1) lacked the Time-At-Depth (TAD) and Timeline (TL) options, which became available in later manufactured models. Consequently, the ten units F10 to F20 had the TAD option, whereas units M13 to F20 also had the TL option. The performance of the Argos system is described in detail by earlier investigators [15], [16]. In this study we summarise the gross horizontal distribution of tagged seals, but a more detailed analysis will be presented elsewhere.

The analyses of diving patterns are based on more than 300 000 recorded dives (Table 2), and the collection of histograms was stratified to four six-hour periods of the day: night (2100-0300) dawn (0300-0900), day (0900-1500), and dusk (1500-2100). The SDRs provided up to seven partly independent measurements of diving behaviour:

1. Depth histograms summarise number of dives per six hour period into six bins with upper limits at 5, 10, 20, 40, 80, and >80 m. The lower limit of the first bin was set at 2 m.
2. Duration histograms summarise numbers of dives into six bins, with the upper limits at: 2, 5, 10, 15, 25 and >25 min. The lower limit of the first bin was set at 0.5 min.
3. Time-at-depth (TAD) bins summarise the time spent at different depths into 8 bins with upper limits set at 2, 5, 20, 40, 60, 80, 100 and 120 m, because the maximum depths in the study areas were 120 m. The upper limit of the first bin was set at 1 m. Only units F10 to F20 had the TAD option (Table 2).
4. Time lines summarize information on whether the unit is at sea or out of water (conductivity sensor dry, >50% of the time) in seventy-two 20-min increments over a 24 hour period. This option gives information on whether or not a seal is hauled out. Only the six units M13 to F20 had this option.
5. The units transmitted every 90 sec “on land”, and every 45 sec “at sea”. This will be referred to as the “Sea/Land Reporter” (SLR). We programmed the transmitters to switch to “on land rate” after 10 consecutive “dry” transmissions. All SDRs were programmed to suspend transmissions after being dry for four hours, and resume transmissions when the seal returned to the water.
6. The transmitters were programmed such that every 15th sent message was a status message, which included the maximum depth measured during the last 24-hour period (midnight-midnight).
7. Time spent at surface was summarised for six-hour periods in the status messages. “At surface times” of seals are defined as the number of seconds (in 90 second increments) during a six-hour period the SDR sensor reads equal or shallower than one meter, or that the conductivity sensor reads “dry”.

**Table 2:** Summary statistics of the information received from the 19 SDRs by month of the year.

Month Estonia	No of dives	No of depth histogr.	No of duration histogr.	No of TAD histogr.^1^	Time lines^1^	Max depth readings^1^	At surface time readings^1^
May	9680	156	132	45	15	29	35
June	21372	302	340	87	27	55	48
July	23197	329	319	73	24	66	52
Aug	18739	298	308	86	19	55	36
Sept	23148	361	382	94	18	69	51
Oct	22892	412	402	99	29	72	53
Nov	33861	541	533	94	19	94	82
Dec	38090	593	579	82	14	110	100
Jan	16603	285	269	57	12	54	51
Feb	8170	113	119	10	1	26	32
March	2191	30	34	0	0	5	6
Total	217943	3388	3416	727	191	635	546
**GOF**							
May	141	2	4	4	4	2	2
June	1886	30	37	40	5	13	13
July	4540	72	66	66	22	17	17
Aug	3797	70	69	60	16	23	23
Sept	3393	65	70	56	15	12	12
Oct	5643	101	93	89	27	21	21
Nov	4769	75	83	87	24	19	19
Dec	5959	98	88	96	29	31	31
Jan	3930	58	61	57	17	14	14
Total	34058	571	571	505	159	152	152
**BB**							
Nov	17526	254	259	250	0	101	101
Dec	16206	227	224	174	0	80	80
Jan	7272	135	135	72	0	35	35
Feb	8626	120	107	18	0	43	43
March	3467	67	71	34	0	25	25
Total	53097	803	796	548	0	284	284
**Grand total**	305098	4762	4783	1780	349	1071	982

1 Depth and duration data from all units, whereas time at depth (TAD) histograms were only sent from units F10 to F20. “Timelines” are available from the six last transmitters (M13-F20). Maximum depth readings over the last 24-hour period, “at surface times”, were extracted from the status messages.

The transmitters which were deployed in the autumn (M1, F2, M3, F10, F11, F12, M13) were programmed to send data signals every day, whereas the remaining 12 (deployed after the moult) transmitted only every second day to save battery power. Maximum daily allowance was set at 500 transmissions and the maximum numbers of transmissions were theoretically 100,000 during the “life span” of batteries. Data on diving behaviour were received from Argos Location Plus System on diskettes and we used the “Sat-pak” programme (Wildlife Computers, version 3.0) to extract relevant data.

### Energy accumulation

All caught seals were weighed using a Salter spring scale (±0.5 kg), but supplementary data of seasonal changes in weights of adult ringed seals was compiled from the seal data base at the Swedish Museum of Natural History. This data is given in Fig. 11.

### Permits

Seals were handled according to national ethical legislation in Estonia (University of Tartu, Estonia), Finland (Finnish Game and Fisheries Institute, Helsinki, Finland), Russia (St. Petersburg State University, St. Petersburg, Russia), and Sweden (Swedish Museum of Natural History, Stockholm, Sweden).
